# *Mycobacterium abscessus *peritonitis associated with laparoscopic gastric banding

**DOI:** 10.1186/1471-2334-13-323

**Published:** 2013-07-15

**Authors:** Hanan I Hakami, Alaa A Alhazmi, Abdulrahman A Alrajhi

**Affiliations:** 1 Section of Infectious Diseases, Department of Medicine, King Faisal Specialist Hospital and Research Centre, Riyadh, Saudi Arabia; 2 Section of General and Minimal Invasive Surgery, Department of Surgery, King Faisal Specialist Hospital and Research Centre, Riyadh, Saudi Arabia

**Keywords:** Laparoscopic gastric banding, Mycobacterium, Peritonitis

## Abstract

**Background:**

*Mycobacterium abscessus* is a rapidly growing *Mycobacterium *that is a common water contaminant in the environment. We report a case of *M. abscessus* infection with band erosion following laparoscopic gastric banding.

**Case presentation:**

A 34-year-old woman developed insidiously progressing abdominal distension over a period of 1 year associated with abdominal pain, fatigue, night sweating and anorexia 4 years after laparoscopic gastric banding for obesity. Investigation revealed significant ascites with caseating granuloma in peritoneal biopsies from which *M. abscessus* was isolated. Band erosion with infection and multiple abdominal adhesions were confirmed during laparoscopic removal of the gastric band. To the best of our knowledge, this is the first reported case of *M. abscessus* infection after laparoscopic gastric banding surgery. We discuss the possible sources of infection, its indolent presentation, and therapeutic challenges.

**Conclusion:**

It is important to consider environmentally acquired infection in patients with signs and symptoms of infection in the presence of surgical prosthesis.

## Background

*Mycobacterium abscessus* is a rapidly growing *Mycobacterium* that is a common water contaminant. Clinical disease due to *M. abscessus* most often presents as chronic lung disease, or as skin, bone or soft-tissue infection following trauma [1-3]. Disseminated disease has been reported in immunocompromised patients [4]. Although nosocomial infections associated with infected prostheses have been reported, such as otitis media following tympanostomy tube placement, peritoneal catheter-related peritonitis, infection after breast augmentation and septic arthritis with joint prosthesis [5-8], there are no reported cases associated with laparoscopic gastric banding devices. Infections with other mycobacteria other than tuberculosis (MOTT) have been reported after gastric banding [9-11]. This report describes a case of *M. abscessus *peritonitis in a patient with a history of laparoscopic gastric banding surgery. We discuss the possible sources and therapeutic challenges of infection, and highlight environmentally acquired infection in patients with signs and symptoms of infection in the presence of surgical prosthesis.

## Case presentation

**Figure 1:**
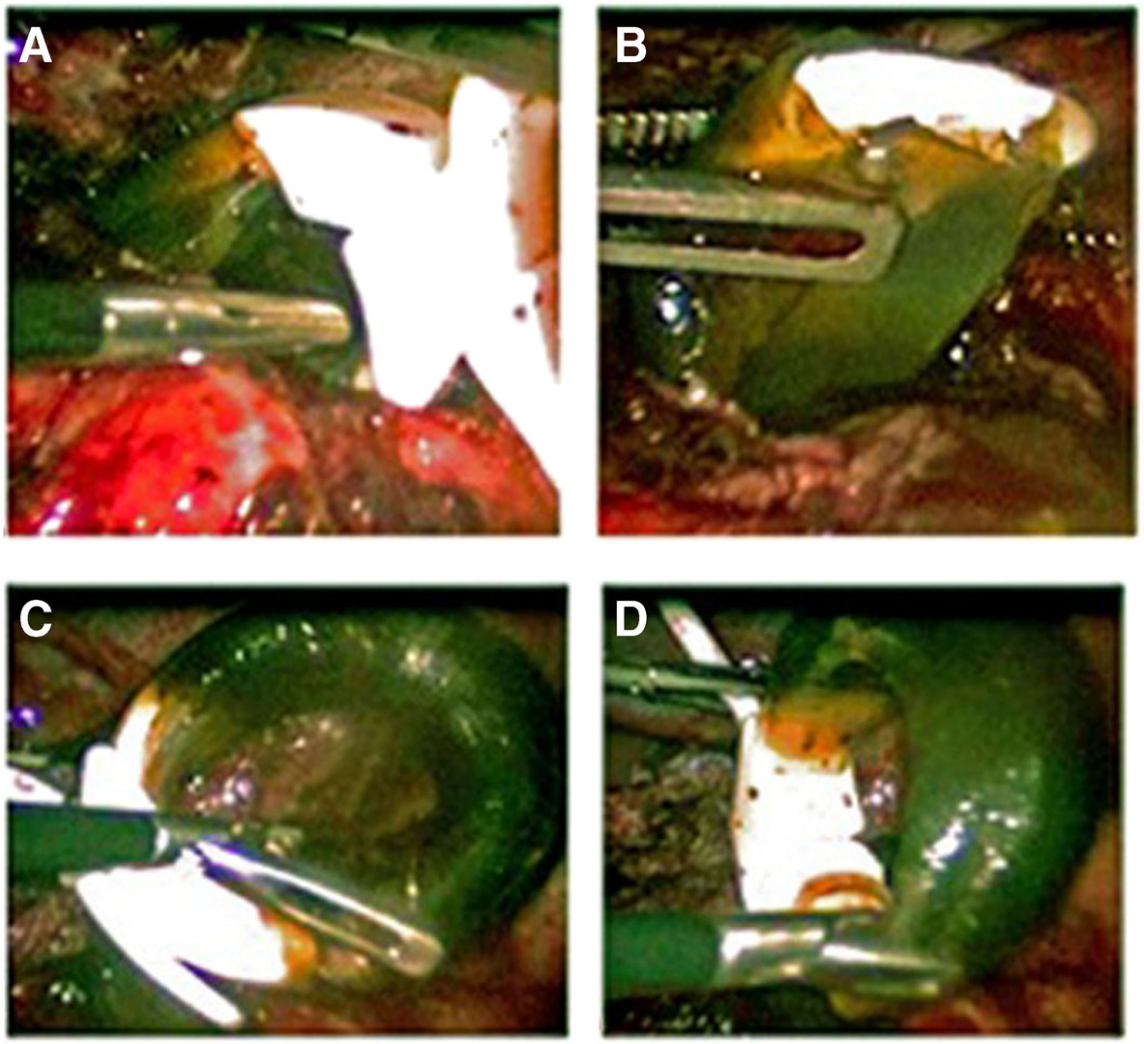
**Infected gastric band. A**: Adhesion around the gastric band. **B**: Fluid from the gastric band. **C**&**D**: Black discoloration of the gastric band.

A 34-year-old woman presented with insidiously progressing abdominal distension of one year’s duration. Review of systems was significant for colicky abdominal pain, fatigue, night sweating and anorexia. The woman had no history of fever, jaundice, tuberculosis contact, weight loss or lower limb swelling. She was not on any medications. Her surgical history included laparoscopic gastric banding surgery for obesity (body mass index = 50; weight, 120 kg) 4 years earlier without complications. The patient had received normal saline injections through an injection port underneath the skin in the first 2 years after surgery to tighten the band. She had lost 50 kg since surgery. She was single, a graphic designer, and a smoker. Her menses were regular and there was no family history of malignancy. The patient’s blood pressure on admission was 110/78 mmHg, her pulse was 82 bpm, her respiratory rate was 18 breaths per minute, and her temperature was 36.8°C. There were no palpable lymph nodes, pallor, jaundice, or other stigmata of chronic liver disease. Abdominal examination revealed a distended soft lax abdomen, a tender epigastrium on deep palpation with no guarding or rigidity, positive shifting dullness, and otherwise normal findings. Complete blood count and hepatic, coagulation and renal profiles were within normal limits and there was no proteinuria. Abdominal ultrasound showed moderate to severe ascites and normal liver size and echogenicity with a patent portal vein. Tumor marker CA 125 was high. Abdominal computed tomography showed large ascites with prominent ovaries bilaterally but no definitive mass or lymphadenopathy. Diagnostic and therapeutic paracentesis was performed, draining 7 l. Fluid analysis showed low serum ascites albumin gradient. Cytology was negative for malignant cells. Laparoscopic peritoneal and ovarian biopsies showed caseating granulomata without malignant cells or acid-fast bacilli. Acid-fast bacilli culture of the ascitic fluid and of peritoneal and ovarian biopsies grew *Mycobacterium* species in both Middlebrook 7H9 Broth (BACTEC MGIT 960 system) and Lowenstein-Jensen after one week of incubation. Colonies appeared smooth and greyish in Lowenstein-Jensen at a temperature of 28°C and 36°C with positive arylsulfatase test and negative ProbeTec ET test. The patient began treatment for rapidly growing *Mycobacterium* infection with clarithromycin, rifampicin, moxifloxacin and ethambutol. The isolate was identified by Line Probe Assay INNO-LiPA MYCOBACTERIA V2 (Innogenetics, Ghent, Belgium) as *M. chelonae complex /abscessus*. The Clinical Microbiology Laboratory at Mayo Clinic used DNA sequencing and real-time PCR, per the Clinical and Laboratory Standards Institute (CLSI) [12],to confirm that the isolate was *M. abscessus,* subspecies *abscessus*. Breakpoint susceptibility testing was performed using the CLSI recommended broth microdilution MIC method with nine drugs (amikacin, tobramycin, trimethoprim-sulfamethoxazole, cefoxitin, imipenem, linezolid, doxycycline/minocycline, clarithromycin and ciprofloxacin) [Table 1]. Breakpoints for tigecycline have not been evaluated by the CLSI for mycobacteria [12]. The isolate was resistant to quinolones and rifampicin, and was susceptible to clarithromycin and amikacin. Rifampicin and moxifloxacin were discontinued and amikacin was started along with ethambutol and clarithromycin. Laparoscopic surgery for gastric band removal was performed. Significant inflammation with dense adhesions surrounding the entire band and black discoloration of the gastric band were noted during the procedure (Figure 1). Adhesiolysiswas performed and the band was removed. Tissue from the inflammation around the band showed granulomata and culture was positive for *M*. *abscessus*. Additional antimicrobials with potential activity against *M*. *abscessus* were added sequentially (imipenem, tigecycline and linezolid), but the patient could not tolerate them for various reasons. After 5 months of clarithromycin and ethambutol treatment supplemented with amikacin for the first 2 months, the patient had an excellent recovery and become completely asymptomatic.

**Table 1:** Broth microdilution interpretive criteria for rapidly growing mycobacteria

	** MIC (μg / mL) for category**		
**Antimicrobial agent**	**Susceptible**	**Intermediate**	**Resistant**
Amikacin	≤ 16	32	≥ 64
Cefoxitin	≤ 16	32–64	≥ 128
Ciprofloxacin	≤ 1	2	≥ 4
Clarithromycin	≤ 2	4	≥ 8
Doxycycline	≤ 1	2–8	≥ 16
Imipenem	≤ 4	8	≥ 16
Linezolid	≤ 8	16	≥ 32
Sulfamethoxazole	≤ 32	-	≥ 64
Tobramycin	≤ 4	8	≥ 16

## Discussion

*M*. *abscessus *is a rapidly growing *Mycobacterium* that is a common water contaminant. Human disease is suspected to result from environmental exposure. Literature review on *Mycobacterium* infections after laparoscopic gastric banding identified three reported cases of *M*. *fortuitum* infections. The first case was a young patient who developed peritonitis within a few days after gastric banding surgery. The other two patients had laparoscopic gastric banding procedures on the same day and in the same operating room [10,11]. *M*. *bolletii* infection was isolated in another reported case after revision of gastric banding [9]. To our knowledge this is the first case of *M*. *abscessus *infection following laparoscopic gastric banding.

We believe that the most likely sources of *Mycobacterium* were the gastric band itself, the surgical environment or contaminated injected saline. Although *M*. *abscessus* was isolated from the inflamed tissue around the gastric band, unfortunately no microbiological culture was sent from either the gastric band itself or the saline. The patient developed abdominal distension 4 years after gastric banding and had three saline band injections. Our patient had a delayed presentation 4 years after surgery, while in the other reported cases of *Mycobacterium*-infected gastric bands, infection occurred within 2 months of surgery.

Our patient developed infection 4 years post-surgery, suggesting an indolent course of this organism. This course is similar to a case of *M*. *abscessus *peritonitis that occurred in the 3rd year after peritoneal catheter placement and after 2 years of persistent isolation of *M*. *abscessus *from the catheter exit site without initial evidence of peritonitis [6].

*M*. *abscessus *isolates are uniformly resistant to standard antituberculosis agents. *M*. *abscessus *is susceptible to clarithromycin (100%), amikacin (90%), cefoxitin (70%), imipenem, linezolid, clofazimine and tigecycline [13]. The role of drug susceptibility testing in the choice of agents for antimicrobial treatment of *M*. *abscessus *remains a subject of debate. There are important discrepancies between drug susceptibility measured *in vitro* and the activity of the drug observed *in vivo*, partly deriving from laboratory technical issues, and no clear standardized treatment method exists. Until the relationship between *in vitro* susceptibility and clinical response of *M*. *abscessus *to antimicrobial drugs is better understood and clarified, antibiotic susceptibility testing of all clinically significant isolates is recommended.

Treatment regimens include clarithromycin for 6 months in cases of skin and soft tissue infections and at least 12 months for pulmonary infections. For serious disseminated infections combination therapy with amikacin plus imipenem or cefoxitin for the first 2–6 weeks usually produces clinical and microbiologic improvement [13]. Regarding *M*. *abscessus *infection and prosthesis, a small number of previously reported cases exist. These include chronic otitis media after tympanostomy tube placement, peritonitis with peritoneal catheter, mastitis after breast augmentation, and septic arthritis with prosthetic joints. Almost all cases required removal of prostheses and antibiotic therapy based on *in vtro* sensitivity for an average duration of 5–6 months, with at least 4–6 weeks of parenteral antibiotics [5-8].

This case highlights an environmentally acquired infection after laparoscopic gastric banding. Physicians need to consider MOTT as a cause of infection in the presence of surgical prosthesis. Treatment should include removal of any infected prosthesis and the use of appropriate antibiotics based on susceptibility testing.

## Conclusion

Our case highlights the potential therapeutic complication of *Mycobacterium* infection in association with laparoscopic gastric banding procedures. When treating patients with signs and symptoms of infection and a history of surgical prosthesis, clinicians should consider MOTT as potential pathogens. Once diagnosed, initial treatment should include appropriate antibiotics selected based on susceptibility results as well as the removal of any infected materials.

## Consent

Written informed consent was obtained from the patient for publication of this case report and any accompanying images. A copy of the written consent is available for review by the Editor of this journal.

## Competing interests

The authors declare that they have no competing interests.

## Authors’ contributions

HH: Wrote the case report and reviewed other reported cases of mycobacteria infection with gastric band. AlA: Contributed to the surgical section of the case report. AbA: Reviewed and contributed to the discussion section. All authors read and approved the final manuscript.

## Authors’ information

Hanan Hakami: Fellow in Infectious Diseases, Department of Medicine, King Faisal Specialist Hospital and Research Centre, Riyadh, Saudi Arabia. Alaa Alhazmi: Consultant of General and Minimal Invasive Surgery, Department of Surgery, King Faisal Specialist Hospital and Research Centre, Riyadh, Saudi Arabia. Abdulrahman Alrajhi: Consultant in Infectious Diseases and Deputy Executive Director, Academic and Training Affairs, King Faisal Specialist Hospital and Research Centre, Riyadh, Saudi Arabia. He authored a chapter on tuberculosis and infectious disease epidemiology. He also obtained a Master’s degree of Public Health from Harvard School of Public Health, Boston, Massachusetts, in International Health/Epidemiology of Infectious Disease.

## Pre-publication history

The pre-publication history for this paper can be accessed here:

http://www.biomedcentral.com/1471-2334/13/323/prepub
